# Biopersistence and Brain Translocation of Aluminum Adjuvants of Vaccines

**DOI:** 10.3389/fneur.2015.00004

**Published:** 2015-02-05

**Authors:** Romain Kroum Gherardi, Housam Eidi, Guillemette Crépeaux, François Jerome Authier, Josette Cadusseau

**Affiliations:** ^1^Faculté de Médecine and Faculté des Sciences et Technologie, INSERM U955 Team 10, Université Paris Est-Créteil, Créteil, France

**Keywords:** alum, vaccine adjuvants, macrophagic myofasciitis, neurotoxicity, genetics, monocytes, CCL2, MCP1

## Abstract

Aluminum oxyhydroxide (alum) is a crystalline compound widely used as an immunological adjuvant of vaccines. Concerns linked to the use of alum particles emerged following recognition of their causative role in the so-called macrophagic myofasciitis (MMF) lesion detected in patients with myalgic encephalomyelitis/chronic fatigue/syndrome. MMF revealed an unexpectedly long-lasting biopersistence of alum within immune cells in presumably susceptible individuals, stressing the previous fundamental misconception of its biodisposition. We previously showed that poorly biodegradable aluminum-coated particles injected into muscle are promptly phagocytosed in muscle and the draining lymph nodes, and can disseminate within phagocytic cells throughout the body and slowly accumulate in brain. This strongly suggests that long-term adjuvant biopersistence within phagocytic cells is a prerequisite for slow brain translocation and delayed neurotoxicity. The understanding of basic mechanisms of particle biopersistence and brain translocation represents a major health challenge, since it could help to define susceptibility factors to develop chronic neurotoxic damage. Biopersistence of alum may be linked to its lysosome-destabilizing effect, which is likely due to direct crystal-induced rupture of phagolysosomal membranes. Macrophages that continuously perceive foreign particles in their cytosol will likely reiterate, with variable interindividual efficiency, a dedicated form of autophagy (xenophagy) until they dispose of alien materials. Successful compartmentalization of particles within double membrane autophagosomes and subsequent fusion with repaired and re-acidified lysosomes will expose alum to lysosomal acidic pH, the sole factor that can solubilize alum particles. Brain translocation of alum particles is linked to a Trojan horse mechanism previously described for infectious particles (HIV, HCV), that obeys to CCL2, signaling the major inflammatory monocyte chemoattractant.

Billions of humans have been vaccinated and marked regression or eradication of several severe infectious diseases was observed. Nowadays, the potential applications of vaccines extend far beyond prevention of infectious diseases, and vaccination is considered to be a most promising weapon against a variety of different conditions. Vaccine safety has been regarded as excellent at the level of the population (1), but adverse effects have also been reported (2).

Concerns about the use of aluminum adjuvants have emerged following (i) recognition of their role at the origin of the so-called macrophagic myofasciitis (MMF) lesion in 2001 (3, 4), which revealed fundamental misconception of their adjuvant effect and pointed out their unexpectedly long-lasting biopersistence (4); and (ii) demonstration of their apparent capacity to migrate in lymphoid organs and then disseminate throughout the body within monocyte-lineage cells and progressively accumulate in the brain (5).

The present paper will review these emerging characteristics of alum adjuvant particles that raise concerns about innocuity of this widely used compound.

## Alum Adjuvants are Lysosome-Destabilizing Particulate Compounds

Adjuvants have been used in vaccines for their ability to enhance the adaptive immune response to a co-administered antigen. Particulate aluminum salts (known as alum) have been the main approved adjuvants for use in human vaccines for more than 80 years (6). They are currently used in vaccines against tetanus, hepatitis A, hepatitis B, human papillomavirus, haemophilus influenzae B, pneumococcal and meningococcal infections, and anthrax. They mainly include aluminum oxyhydroxide, a crystalline compound, aluminum hydroxyphosphate, and amorphous aluminum phosphate. Alum is able to adsorb vaccine antigens on its surface. The strongest adsorption phenomenon results from ligand exchange, which involves the replacement of a surface hydroxyl on the adjuvant by a terminal phosphate group of the antigen (7).

Alum induces strong innate immune responses at the site of injection, as assessed by an influx of neutrophils, monocyte/macrophages, eosinophils, and MHC-II + antigen presenting cells, mainly dendritic cells (DCs) (8). Muscle-resident macrophages mainly located in fascias are among the first cells to sense disturbance in muscle homeostasis (9). They alert the immune system through local production of chemokines, and recruit other myeloid cells, like neutrophils, and inflammatory monocytes that differentiate into inflammatory DCs (9). Specialized for antigen uptake, monocyte-derived inflammatory DCs have an immature phenotype in the muscle. However, they migrate to the lymph node T-cell paracortex upon contact with tissue debris or foreign material, and arrive there as mature cells expressing costimulatory molecules (10). Inflammatory DCs may be crucial for the alum adjuvant activity as assessed by selective depletion studies (11), but eosinophils also appear to play an important role (12).

Alum has been long believed to ensure a long-lasting immune response through formation of a depot slowly releasing the antigen under the influence of the interstitial fluid (13, 14). The view that the injected adjuvant remains extra-cellular has been challenged by muscle biopsy findings in immunized patients (4). In contrast to ancient belief, alum particles are avidly taken up by phagocytic cells (15). The strong binding of antigen to alum particles increases antigen uptake by DCs, reduces antigen degradation, and sustains antigen presentation *in vitro* (16). Macrophage survival may also be promoted by alum particle uptake (17). Alum injection induces *in vivo* the formation of persistent alum-induced granuloma at site of previous immunization (4, 18, 19). However, good immunization does not require local alum persistence, since no decrease of antigen-specific T- and B-cell responses were observed in case of removal of the injection site as early as 2 h after injection (20).

In spite of their long usage, the literature has pointed out that the adjuvanticity mechanisms of aluminum salts remain basically unknown despite most active investigation in the field in recent years (21, 22). Alum is deficient at initiating cell-mediated immunity and skews the immune response toward a T-helper type 2 (Th2) response associated with strong production of IL-4 and the IgG1 antibody subtype (23). Concerning the mechanisms of alum adjuvanticity, several explanations have been proposed, most of them being subsequently challenged (24). Notably, the NLRP3 inflammasome was shown to be strongly activated by alum (25, 26), but this finally appeared unessential to the adjuvant effect (27, 28). It remains true, however, that aluminum hydroxide and other crystals such as silica, urate sodium, and asbestos, strongly induce NLRP3 activation, IL1b release, and activation of the downstream inflammatory cascade. More recently, alternate models for alum-mediated immunity have been proposed on the basis of the link of alum adjuvant effects and the release of non-cytokine biomolecules, including uric acid (29), double-stranded DNA (30), and prostaglandin E2 (31). The specificity of crystal-induced signaling pathways has been proposed to explain why aluminum hydroxide particles exhibit a much more irritating effect than soluble aluminum (32). Consistently, alum crystals bind to and aggress the plasma membrane lipid bilayer (33), destabilizes lysosomes that degrade endocytosed, phagocytosed, or autophagocytosed materials (34, 35), and play important role in immunity. Highly controlled antigen processing functions of DCs use lysosomal proteases and pH changes optimal for the generation of peptides, rather than complete protein degradation (36). It is known that limitation of lysosomal proteolysis of antigenic proteins increases antigen presentation and immunogenicity (37), and that the stability of peptide:MHCII complexes allowing their accumulation on the DC surface is enhanced by lysosome activity inhibition (38). Alum adjuvant mechanisms may thus involve alum-induced blockade of lysosomes. Alum lysosomal destabilization remains still uncertain, but the physical rupture of the membrane may be directly caused by the crystalline structure of alum itself (39).

## MMF is a Biomarker Assessing Long-Term Alum Biopersistence in a Given Individual

In 1998, several French myopathologists described MMF as an emerging condition of unknown cause characterized by a pathognomonic lesion in muscle biopsy mixing large macrophages with submicron to micron-sized agglomerates of nanocrystals in their cytoplasm and lymphocytic infiltrates (3), distinct from other histiocytic diseases and always detected in the deltoid muscle of adults (40). Cytoplasmic inclusions were constantly found, surrounded or not by altered lysosomal membranes, and contained aluminum (4). Their crystalline structure was characteristic of aluminum hydroxide, and no exposure to aluminum other than that conferred by a prior immunization (100%) could be detected (4). It is now clear that the rapid emergence of MMF in France reflected the combination of (i) the replacement of the subcutaneous (s.c.) by the intramuscular (i.m.) route for vaccine injections in the early 1990s; (ii) the large-scale campaign of primo-vaccination of French adults against hepatitis B in the mid 1990s; and (iii) the preferential choice of the deltoid muscle for routine muscle biopsy in France, contrasting with the preferential use of the biceps brachialis and quadriceps muscles in other countries. Alum-containing vaccines may also induce skin pseudo-lymphoma in humans (41), and fibrosarcoma in cats (42).

Macrophagic myofasciitis has been reproduced experimentally by i.m. vaccination in mice, rats, and monkeys (4, 18, 19). The experimental lesion invariably shrinks over time (19), and, in monkeys, it begins to disappear completely from the muscle between 6 and 12 months after a DTP injection corresponding to 14- to 21-fold the human DTP-equivalent dose of alum (18).

Because of the unethical character of muscle biopsy in asymptomatic individuals, whether or not longstanding MMF may be commonly present in a hidden form in healthy individuals could not be directly determined. This seems very unlikely, however, as shown in a recent review of 130 consecutive deltoid muscle biopsies performed for diagnostic purposes in myalgic patients previously immunized with alum-containing vaccines. This study revealed that most alum receivers do not have long-lasting MMF. This could be reliably assessed whereas age, sex ratio, number of alum-adjuvanted injections, and delays elapsed from the last injection to deltoid muscle biopsy were similar in the MMF and non-MMF groups (43). This refutes non-documented belief that every vaccinee may have long-standing MMF lesions when biopsy is performed in the deltoid muscle (44). In addition, MMF and non-MMF patients had clinical differences as developed below.

In light of experimental models, it is important to check the individual vaccine record in each patient to assess the “unusually persistent” character of MMF. In a recent evaluation of 583 patients collected from 1994 to 2012 (45), the median time elapsed between the last alum administration and the biopsy was 65 months. Compared to our previous reports, this time had gradually increased from 36 months in 2001, i.e., shortly after the peak of French adult immunization, to 53 months in 2003 (46). An average number of 5.3 alum-containing shots had been administered during the 10 years prior to biopsy detecting MMF, mainly corresponding to vaccinations against hepatitis B (89.7%), tetanus (42.2%), and hepatitis A (8.8%). In practice, we consider that the MMF is unusually persistent when time elapsed from last immunization to the MMF detection exceeds 18 months. It is important to consider this point in young children who receive multiple vaccine injections in the first year of life, thus increasing the risk of coincidental association between a constitutive muscle disease and MMF detected in the quadriceps muscle used for pediatric immunizations. If the risk of such coincidental associations also potentially exists in adults, it is low in practice. For example, adult patients combining MMF and hereditary muscle disease is extremely rare, despite the intense immunization program of patients with muscular dystrophy.

Animal studies indicate that alum-induced granulomatous lesions considerably vary in size according to the genetic background (19), and the initial hypothesis made by WHO that MMF may reflect some individual inability to clear out alum from the body remains valid (47). In summary, the long-lasting MMF lesion should be considered as a biomarker assessing unusually long-term biopersistence of alum in affected individuals.

## Patients with MMF at Biopsy Suffer Myalgic Encephalomyelitis/Chronic Fatigue Syndrome

Macrophagic myofasciitis is typically detected in patients with diffuse myalgias and chronic fatigue, as shown in both the French series (46) and the recently published series of 16 patients (48).

In both series, most patients are women (70–80%) with a mean age of 45 years at the time of the biopsy, that typically complain of myalgias, with or without arthralgia, and disabling chronic fatigue. The onset of these symptoms is typically delayed from the immunization.

Strong statistical association between myalgias and MMF was detected by general survey in different French neuromuscular centers (myalgias in 90% of patients with MMF vs. 44% without MMF, *p* < 0.0001) (4). Onset of myalgia may follow exercise. They usually begin in the lower limbs, and not at the site of previous immunization from 0.5 to 84 months in the French patients and 3 to 192 months in Portuguese patients. They gradually extend toward the top of the body, affect the paravertebral muscles, and become diffuse (46). Myopathic electromyogram and elevation of creatine kinase (CK) are, respectively, observed in less than half of patients. Comparison of myalgic vaccinees with and without MMF at deltoid muscle biopsy showed significant differences: patients with MMF rarely had fibromyalgia (the required 11 tender points of the ACR 1990 criteria for fibromyalgia present in 16.6 vs. 55.5%, *p* < 0.04), and more often had delayed evoked potentials suggestive of CNS demyelination (38.5 vs. 5.7%, *p* < 0.01) (43), which does not support coincidental association.

Chronic fatigue is another important symptom (48, 49). A case–control study conducted under the aegis of the French regulatory agency AFSSAPS yielded chronic fatigue as both significantly more frequent and more severe in patients with MMF compared to those without MMF in the deltoid muscle (http://ansm.sante.fr/var/ansm_site/storage/original/application/030593fa4e393af7cec8ff7092832215.pdf).

Cognitive alterations further assess CNS involvement that are disabling though often not detected by routine examination. Patients complain of memory loss, foggy brain, and mood changes. Cognitive tests almost constantly show alterations suggestive of organic cortico-subcortical impairment, impacting visual memory, working memory, and dichotic listening (50). These deficits usually remain stable with time (51).

Taken together, chronic muscle pain, chronic fatigue, and cognitive dysfunction are consistent with the so-called myalgic encephalomyelitis/chronic fatigue syndrome (ME/CFS) and about 50% of MMF patients meet international criteria for ME/CFS (48, 49). ME/CFS is a severe, complex, acquired illness classified as a neurological disorder in the WHO International Classification of Diseases since 1969 (ICD 10 G93.3), distinct from fibromyalgia and psychasthenia, which are classified as musculoskeletal (M79.7) and psychiatric (F48.8) disorders, respectively. International studies have estimated the prevalence of ME/CFS between 0.4 and 2.6% of the population, with a total annual cost burden to society of approximately $18.7–$24.0 billion in the USA (52). Symptoms of ME/CSF are closely similar to the post-infective chronic fatigue syndrome (53). The underlying cause of ME/CSF is currently unknown, but the illness is thought to be triggered by an abnormal immune response to an infectious or toxic agent, that results in chronic immune activation (54). Notably, ME/CFS patients have increased risk of developing diffuse large B-cell lymphoma and marginal zone B-cell lymphoma (55). Such a public health burden deserves continued efforts to investigate possible causes and to understand the pathological mechanisms of CFS.

## Phagocytes Transport Alum Particles to the Lymphoid Organs and then to the Brain

The conceptual link between long-term persistence of alum particles within macrophages at the site of previous immunization, and the occurrence of adverse systemic events, in particular neurological ones, has long remained an unsolved question. Aluminum has long been identified as a neurotoxic metal, affecting memory, cognition and psychomotor control, altering neurotransmission and synaptic activity, damaging the blood–brain barrier (BBB), exerting pro-oxidant effects, activating microglia and neuroinflammation, depressing the cerebral glucose metabolism and mitochondrial functions, interfering with transcriptional activity, and promoting beta-amyloid and neurofilament aggregation (56). In addition, alum particles impact the immune system through their adjuvant effect and by many other means. They adsorb vaccine antigens on their surface, which protect them from proteolysis thus forming a persistently immunogenic pseudo-pathogen (57). Alum particles may also bind undesirable residual products inherent to vaccine production procedures, as shown for HPV DNA sequences (58) or yeast proteins (59) that may be potentially hazardous (60). Finally, alum particles can directly induce allergy (61, 62) as other metals (63).

Concerns about long-term biopersistence of alum largely depend on the ability of alum particles to reach and exert toxicity in remote organs. This ability has been suggested by several studies (64–67). The reference study on aluminum hydroxide biodisposition used isotopic ^26^Al-enriched alum injected in the rabbit muscle: ^26^Al was weakly eliminated in the urine (6% on day 28) and was detected in lymph nodes, spleen, liver, and brain (13). Whether ^26^Al was still in particulate form or in soluble form was not explored. The fate of particulate material was explored in mice by our team. We successively performed i.m. injections of alum-containing vaccine, fluorescent latex beads, and fluorescent nanohybrids coated with precipitated alum (5). These materials were quickly captured by macrophages, a large proportion of which cleaved the injected muscle, mainly within immune cells, reaching the draining lymph nodes. Particle-laden cells then escaped the lymphatic system to reach the blood circulation, presumably via the thoracic duct. In so-doing, they were able to reach distant organs such as the spleen and liver and, much slowly, the brain. Recombinant chemokine injection and the use of genetically modified mice showed that systemic biodistribution of particles crucially depends on the monocyte chemoattractant MCP-1/CCL2. Into the brain, particles were mainly found in microglial cells. In accordance with good overall tolerance of alum, brain penetration was extremely low in normal conditions. However, brain translocation was significantly increased in case of altered BBB or after systemic and/or cerebral increase of the MCP-1/CCL2 signaling (5). Expression of this chemokine is subjected to significant interindividual variations related to age, genetic, and environmental factors. We have identified selective increase of circulating MCP-1/CCL2 in CFS/ME patients with MMF (45). The imbalance between the huge number of vaccinated individuals and the relatively low number of MMF cases suggests crucial involvement of individual susceptibility factors in intolerance to alum. Genetically driven MCP-1/CCL2 production might represent one of these factors (5).

Thus alum and other poorly biodegradable materials taken up at the periphery by phagocytes circulate in the lymphatic and blood circulation and can enter the brain using a Trojan horse mechanism similar to that used by infectious particles (68, 69). Previous experiments have shown that alum administration can cause CNS dysfunction and damage (70–72), casting doubts on the exact level of alum safety (73).

## The Concept of ASIA

Many CNS diseases likely result from gene–environment interactions. Some of them, such as idiopathic ME/SFC (74) and multiple sclerosis (MS) (75), have been previously associated with aluminum overload. An increased risk of developing MS in the long-term after alum-containing vaccine administration has been also reported (76, 77), and remains the subject of fierce debate.

Notably, about 10% of our MMF patients had concurrent MS-like disease (78), an additional 5–10% had another autoimmune disease, such as thyroiditis and diffuse inflammatory myopathies, and the remaining patients occasionally had low titers of various autoantibodies (46).

Yehuda Shoenfeld had delineated the “autoimmune (autoinflammatory) syndrome induced by adjuvants” (ASIA)(79), acknowledging that various combinations of (i) specific autoimmune diseases identified by well-established criteria, (ii) less-specific symptoms, such as myalgia, arthralgia, chronic fatigue, and cognitive impairment (the combination of which defines ME/CFS); and (iii) the appearance of circulating autoantibodies, can occur after exposure to a variety of chemical or natural products with immunological adjuvant properties. Discussion of the ASIA is very useful since it may alert physicians, when they encounter the above-mentioned symptoms, to check for prior vaccinations, and may help them to put a name on such conditions.

Symptoms associated with MMF are strikingly similar to those described as the Gulf war syndrome (GWS), a condition strongly associated with the administration of multiple vaccinations to soldiers (80, 81), especially the anthrax vaccine that contains alum, capable of inducing MMF (82), and possibly squalene (83). On these grounds, we proposed to delineate a vaccine adjuvant syndrome (84). Yehuda Shoenfeld reasoned similarly but added to GWS and MMF, his own experience on siliconosis, a disease complex observed in patients with leaky breast silicone implants attributed to deleterious adjuvanticity of silicone particles (85, 86). In so-doing, he enlarged the causal relationship to any compound with adjuvant properties. ASIA major and minor diagnostic criteria still need international validation but the ASIA concept already caught the attention of the international human and veterinary medical community, pointing out a need in the field (87, 88).

## A Lot Must be Done to Understand How, in Certain Individuals, Alum-Containing Vaccines may Become Insidiously Unsafe

Alum has been used for decades to levels considered as an acceptable compromise between its role of adjuvant and its toxic effects by the industry and the regulatory agencies. However, the MMF story revealed several gaps in the knowledge on alum particles, including their exact mechanisms of action, their fate after injection, their systemic dissemination, and their safety on the long-term. Efforts have been done in the last years to develop novel adjuvants, but attempts to seriously examine safety concerns raised by the bio-persistent character and brain accumulation of alum particles have not been made.

The main questions that should be addressed concerning alum safety problems are listed in Table 1. It is important to look for genetic susceptibility factors that could explain why a given individual will appear intolerant to alum-containing vaccines whereas the vast majority of individuals vaccinated with the same vaccines remain healthy. Some patients with MMF are of the HLA-DRB1*01group, which is associated with an increased risk to develop autoimmune diseases (89). Genetic factors influencing alum biodistribution were also investigated. In keeping with experimental evidence that the CCL2/MCP-1 chemokine signaling governs brain translocation of phagocytosed particles (5), and that CCL2/MCP-1 serum levels are selectively increased in patients with MMF (45), genotyping of 252 symptomatic MMF patients and 516 healthy controls for 4 single nucleotide polymorphisms (SNPs) localized in the CCL2 gene showed that the AG haplotype of the SNP rs3760396C(−927G > C) was associated with a slightly increased risk for disease (5). Interestingly, the rs 3760396 C allele is associated with a higher level of expression of CCL2 *in vitro* as assessed by transfection (90). These preliminary results deserve further investigations. Another axis of research consists in attempts to detect if subtle genetically determined defects in the cell machinery used to clear out particles, namely autophagy (91), could contribute to the long-standing biopersistence of alum particles, as previously reported to explain intracellular persistence of intestinal pathogens in Crohn’s disease (92). Cells coping with microbes use a dedicated form of autophagy termed “xenophagy” as a host defense mechanism to engulf and degrade intracellular pathogens. The same holds true for inert particles subjected to phagocytosis/endocytosis (93). As mentioned above, crystal particles are likely toxic to membranes, which may destabilize phagosomes and lysosomes, trigger inflammasome assembly, and impede the autophagy pathways (32–35, 39). However, crystal particles instead of killing macrophages promote their survival (17). Thus, macrophages will continuously perceive as foreign particles in their cytosol, just like senescent organelles or bacteria, and will likely reiterate the autophagic process until they dispose of alien materials. The process includes compartmentalization of particles within double membrane autophagosomes and subsequent fusion with repaired and re-acidified lysosomes, exposing antigen-bound alum particles to lysosomal acidic pH, the sole factor that can solubilize alum crystal and acid hydrolases that will degrade the antigen. The process involves a conserved pathway in which particles decorated by ubiquitinated proteins, recruit the adaptor protein p62/SQSTM1 (sequestosome 1), which targets the whole to the autophagosome through binding to the autophagosomal membrane protein LC3/Atg8 (94, 95). Autophagosomes formation also involves other Atg molecules, such as the high molecular weight complex (Atg12–Atg5–Atg16L), Atg7, and many others, and is regulated by IRGM (immunity-related GTPase family-M1). The autophagosome external membrane eventually fuses with lysosomes. Genes of all molecules of the autophagy pathway are subjected to variations that are currently screened in patients with MMF.

**Table 1:** **Main unsolved questions linked to alum adjuvants toxic effects**.

**WHAT IS THE MOST TOXIC?**
Al^3+^metal toxicity (or allergy to Al)
Particle toxicity due to elementary nanoparticles, e.g., mitochondrial toxicity, or to the micronic agglomerates they form, e.g., proinflammatory effects
Immune reactions against biopersistent biomolecules adsorbed on alum, and protected from degradation until complete particle solubilization (vaccine antigen or trace residual DNA sequences linked to vaccine production, or even self-antigens adsorbed on alum at time of injection-induced muscle necrosis)
**WHAT FACTORS CONTRIBUTE TO BIOPERSISTENCE?**
The quantity administered
Adsorbed molecules impeding extracellular solubilization and/or favoring phagocytosis of alum particles
Crystalline structure of the adjuvant damaging lipid bilayers (e.g., lysosomes)
**WHAT FACTORS CONTRIBUTE TO BRAIN TRANSLOCATION?**
Al^3+^ ion transport by transferrin (receptors present in CNS increase with iron deficiency)
Direct BBB damage by alum particles (proportion and kinetics in the circulation are unknown)
Monocyte cell transport of particles (the MCP1/CCL2-dependent Troian horse mechanism is increased in case of altered BBB and/or neuroinflammation)
**WHAT ARE THE SUSCEPTIBILITY FACTORS?**
Individual environment (other exposures to Al, exposure to other metals, exposure to other particles, chronic viral infection)
Age of immunization, including early age (low body weight, immature BBB, early neurodevelopmental stage) and old age (increased MCP-1/CCL2 production, progressive BBB weakness, hidden neuropathological processes)
Genetic factors impacting either immunologic responses (e.g., HLA genotypes) or intracellular persistence of particles (xeno/autophagy genes), or neuromigration (chimiokines and other inflammation genes)

## Conflict of Interest Statement

The authors declare that the research was conducted in the absence of any commercial or financial relationships that could be construed as a potential conflict of interest.

## References

[1] Moxon ER Siegriest CA. The next decade of vaccines: societal and scientific challenges. Lancet (2011) 378:348–59. 10.1016/S0140-6736(11)60407-8 21664685

[2] Agmon-Levin N Paz Z Israeli E Shoenfeld Y Vaccines and autoimmunity. Nat Rev Rheumatol (2009) 5(11):648–52 10.1038/nrrheum.2009.196 19865091

[3] Gherardi RK Coquet M Chérin P Authier FJ Laforêt P Bélec L Macrophagic myofasciitis: an emerging entity. Lancet (1998) 352(9125):347–52. 10.1016/S0140-6736(98)02326-5 9717921

[4] Gherardi RK Coquet M Cherin P Belec L Moretto P Dreyfus PA Macrophagic myofasciitis lesions assess long-term persistence of vaccinederived aluminum hydroxide in muscle. Brain (2001) 124:1821–31. 10.1093/brain/124.9.1821 11522584

[5] Khan Z Combadiere C Authier FJ Itier V Lux F Exley C Slow CCL2-dependent translocation of biopersistent particles from muscle to brain. BMC Med (2013) 11:99. 10.1186/1741-7015-11-99 23557144 PMC3616851

[6] Glenny A Pope C Waddington H Wallace U Immunological notes. XVII. The antigenic value of toxoid precipitated by potassium alum. J Pathol Bacteriol (1926) 26:38–9.

[7] Lu F Boutselis I Borch RF Hogenesch H. Control of antigen-binding to aluminum adjuvants and the immune response with a novel phosphonate linker. Vaccine (2013) 13:946–8. 10.1016/j.vaccine.2013.07.019 23887038

[8] Lu F Hogenesch H. Kinetics of the inflammatory response following intramuscular injection of aluminum adjuvant. Vaccine (2013) 31(37):3979–86. 10.1016/j.vaccine.2013.05.107 23770306

[9] Brigitte M Schilte C Plonquet A Baba-Amer Y Henri A Charlier C Muscle resident macrophages control the immune cell reaction in a mouse model of notexin-induced myoinjury. Arthritis Rheum (2010) 62:268–79. 10.1002/art.27183 20039420

[10] Liao H Franck E Fréret M Adriouch S Baba-Amer Y Authier FJ Myoinjury transiently activates muscle antigen-specific CD8+ T cells in lymph nodes in a mouse model. Arthritis Rheum (2012) 64(10):3441–51. 10.1002/art.34551 22674045

[11] Kool M Soullié T van Nimwegen M Willart MA Muskens F Jung S Alum adjuvant boosts adaptive immunity by inducing uric acid and activating inflammatory dendritic cells. J Exp Med (2008) 205:869–82. 10.1084/jem.20071087 18362170 PMC2292225

[12] Wang HB Weller PF. Pivotal advance: eosinophils mediate early alum adjuvant-elicited B cell priming and IgM production. J Leukoc Biol (2008) 83:817–21. 10.1189/jlb.0607392 18192490 PMC2735468

[13] Flarend RE Hem SL White JL Elmore D Suckow MA Rudy AC In vivo absorption of aluminum-containing vaccine adjuvants using ^26^Al. Vaccine (1997) 15:1314–8. 10.1016/S0264-410X(97)00041-8 9302736

[14] Shi Y HogenEsch H Hem SL. Change in the degree of adsorption of proteins by aluminum-containing adjuvants following exposure to interstitial fluid: freshly prepared and aged model vaccines. Vaccine (2001) 20:80–5. 10.1016/S0264-410X(01)00313-9 11567749

[15] Morefield GL Sokolovska A Jiang D HogenEsch H Robinson JP Hem SL. Role of aluminum-containing adjuvants in antigen internalization by dendritic cells in vitro. Vaccine (2005) 23:1588–95. 10.1016/j.vaccine.2004.07.050 15694511

[16] Ghimire TR Benson RA Garside P Brewer JM. Alum increases antigen uptake, reduces antigen degradation and sustains antigen presentation by DCs in vitro. Immunol Lett (2012) 147(1–2):55–62. 10.1016/j.imlet.2012.06.002 22732235 PMC3477319

[17] Hamilton JA Byrne R Whitty G. Particulate adjuvants can induce macrophage survival, DNA synthesis, and a synergistic, proliferative response to GM-CSF and CSF-1. J Leukoc Biol (2000) 67:226–32. 10670584

[18] Verdier F Burnett R Michelet-Habchi C Moretto P Fievet-Groyne F Sauzeat E. Aluminum assay and evaluation of the local reaction at several time points after intramuscular administration of aluminum containing vaccines in the *Cynomolgus* monkey. Vaccine (2005) 23:1359–67. 10.1016/j.vaccine.2004.09.012 15661384

[19] Authier FJ Sauvat S Christov C Chariot P Raisbeck G Poron MF AlOH3-adjuvanted vaccine-induced macrophagic myofasciitis in rats is influenced by the genetic background. Neuromuscul Disord (2006) 16:347–52. 10.1016/j.nmd.2006.02.004 16616846

[20] Hutchison S Benson RA Gibson VB Pollock AH Garside P Brewer JM. Antigen depot is not required for alum adjuvanticity. FASEB J (2012) 26:1272–9. 10.1096/fj.11-184556 22106367 PMC3289510

[21] Exley C Swarbrick L Gherardi RK Authier FJ. A role for the body burden of aluminum in vaccine-associated macrophagic myofasciitis and chronic fatigue syndrome. Med Hypotheses (2009) 72:135–9. 10.1016/j.mehy.2008.09.040 19004564

[22] Exley C Siesjo P Eriksson H. The immunobiology of aluminum adjuvants: how do they really work? Trends Immunol (2010) 31:103–9. 10.1016/j.it.2009.12.009 20153253

[23] Ulanova M Tarkowski A Hahn-Zoric M Hanson LA. The common vaccine adjuvant aluminum hydroxide up-regulates accessory properties of human monocytes via an interleukin-4-dependent mechanism. Infect Immun (2001) 69:1151–9. 10.1128/IAI.69.2.1151-1159.2001 11160013 PMC97997

[24] McKee AS Munks MW MacLeod MK Fleenor CJ Van Rooijen N Kappler JW Alum induces innate immune responses through macrophage and mast cell sensors, but these sensors are not required for alum to act as an adjuvant for specific immunity. J Immunol (2009) 183:4403–14. 10.4049/jimmunol.0900164 19734227 PMC2912728

[25] Eisenbarth SC Colegio OR O’Connor W Sutterwala FS Flavell RA Crucial role for the Nalp3 inflammasome in the immunostimulatory properties of aluminum adjuvants. Nature (2008) 453:1122–6 10.1038/nature06939 18496530 PMC4804622

[26] Li H Willingham SB Ting JP Re F Cutting edge. Inflammasome activation by alum and alum’s adjuvant effect are mediated by NLRP3. J Immunol (2008) 181:17–21 10.1111/j.1365-2567.2007.02774.x 18566365 PMC2587213

[27] Franchi L Nunez G. The Nlrp3 inflammasome is critical for aluminum hydroxide-mediated IL-1beta secretion but dispensable for adjuvant activity. Eur J Immunol (2008) 38:2085–9. 10.1002/eji.200838549 18624356 PMC2759997

[28] Spreafico R Ricciardi-Castagnoli P Mortellaro A. The controversial relationship between NLRP3, alum, danger signals and the next generation adjuvants. Eur J Immunol (2010) 40:638–42. 10.1002/eji.200940039 20201020

[29] Kool M Petrilli V De Smedt T Rolaz A Hammad H van Nimwegen M Cutting edge: alum adjuvant stimulates inflammatory dendritic cells through activation of the NALP3 inflammasome. J Immunol (2008) 181:3755–9. 10.4049/jimmunol.181.6.3755 18768827

[30] Marichal T Ohata K Bedoret D Mesnil C Sabatel C Kobiyama K DNA released from dying host cells mediates aluminum adjuvant activity. Nat Med (2011) 17:996–1002. 10.1038/nm.2403 21765404

[31] Kuroda E Ishii KJ Uematsu S Ohata K Coban C Akira S Silica crystals and aluminum salts regulate the production of prostaglandin in macrophages via NALP3 inflammasome-independent mechanisms. Immunity (2011) 34(4):514–26. 10.1016/j.immuni.2011.03.019 21497116

[32] Shi Y. To forge a solid immune recognition. Protein Cell (2012) 3:564–70. 10.1007/s13238-012-2933-5 22717983 PMC4875360

[33] Flach TL Ng G Hari A Desrosiers MD Zhang P Ward SM Alum interaction with dendritic cell membrane lipids is essential for its adjuvanticity. Nat Med (2011) 17:479–87. 10.1038/nm.2306 21399646

[34] Hornung V Bauernfeind F Halle A Samstad EO Kono H Rock KL Silica crystals and aluminum salts activate the NALP3 inflammasome through phagosomal destabilization. Nat Immunol (2008) 9:847–56 10.1038/ni.1631 18604214 PMC2834784

[35] Lima H Jr Jacobson LS Goldberg MF Chandran K Diaz-Griffero F Lisanti MP Role of lysosome rupture in controlling Nlrp3 signaling and necrotic cell death. Cell Cycle (2013) 12(12):1868–78. 10.4161/cc.24903 23708522 PMC3735701

[36] Trombetta ES Ebersold M Garrett W Pypaert M Mellman I. Activation of lysosomal function during dendritic cell maturation. Science (2003) 299:1400–3. 10.1126/science.1080106 12610307

[37] Delamarre L Couture R Mellman I Trombetta ES. Enhancing immunogenicity by limiting susceptibility to lysosomal proteolysis. J Exp Med (2006) 203:2049–55. 10.1084/jem.20052442 16908625 PMC2118388

[38] Shin JS Ebersold M Pypaert M Delamarre L Hartley A Mellman I. Surface expression of MHC class II in dendritic cells is controlled by regulated ubiquitination. Nature (2006) 444:115–8. 10.1038/nature05261 17051151

[39] Kang SJ Locksley RM. The inflammasome and alum-mediated adjuvanticity. F1000 Biol Rep (2009) 1:15. 10.3410/B1-15 20948671 PMC2920669

[40] Bassez G Authier FJ Lechapt-Zalcman E Delfau-Larue MH Plonquet A Coquet M Inflammatory myopathy with abundant macrophages (IMAM): a condition distinct from macrophagic myofasciitis, sharing similarities with cytophagic histiocytic panniculitis. J Neuropathol Exp Neurol (2003) 62:464–74. 12769186 10.1093/jnen/62.5.464

[41] Maubec E Pinquier L Viguier M Caux F Amsler E Aractingi S Vaccination-induced cutaneous pseudolymphoma. J Am Acad Dermatol (2005) 52(4):623–9. 10.1016/j.jaad.2004.12.021 15793512

[42] Madewell BR Griffey SM McEntee MC Leppert VJ Munn RJ. Feline vaccine-associated fibrosarcoma: an ultrastructural study of 20 tumors (1996-1999). Vet Pathol (2001) 38(2):196–202. 10.1354/vp.38-2-196 11280376

[43] Ragunathan-Thangarajah N Le Beller C Boutouyrie P Bassez G Laurent S Authier FJ. Distinctive clinical features in arthromyalgic patients with and without aluminum hydroxide-induced macrophagic myofasciitis: an exploratory study. J Inorg Biochem (2013) 128:262–6. 10.1016/j.jinorgbio.2013.07.020 23921285

[44] Papo T Macrophagic myofasciitis: focal or systemic? Joint Bone Spine (2003) 70:242–5 10.1016/S1297-319X(03)00093-9 12951304

[45] Cadusseau J Ragunathan-Thangarajah N Surenaud M Hue S Authier FJ Gherardi RK. Selective elevation of circulating CCL2/MCP1 levels in patients with longstanding post-vaccinal macrophagic myofasciitis and ASIA. Curr Med Chem (2014) 21:511–7. 10.2174/09298673113206660287 24083602 PMC5103034

[46] Gherardi RK Authier FJ. Aluminum inclusion macrophagic myofasciitis: a recently identified condition. Immunol Allergy Clin North Am (2003) 23:699–712. 10.1016/S0889-8561(03)00095-X 14753387

[47] World Health Organization Vaccine Safety Advisory Committee. Macrophagic myofasciitis and aluminum-containing vaccines. Wkly Epidemiol Rec (1999) 74:338–40.

[48] Santiago T Rebelo O Negrão L Matos A. Macrophagic myofasciitis and vaccination: consequence or coincidence? Rheumatol Int (2015) 35(1):189–92. 10.1007/s00296-014-3065-4 24923906

[49] Authier FJ Sauvat S Champey J Drogou I Coquet M Gherardi R Chronic fatigue syndrome in patients with macrophagic myofasciitis. Arthritis Rheum (2003) 48:569–70 10.1002/art.10740 12571868

[50] Couette M Boisse MF Maison P Brugieres P Cesaro P Chevalier X Long-term persistence of vaccine-derived aluminum hydroxide is associated with chronic cognitive dysfunction. J Inorg Biochem (2009) 103:1571–8. 10.1016/j.jinorgbio.2009.08.005 19748679

[51] Passeri E Villa C Couette M Itti E Brugieres P Cesaro P Long-term follow-up of cognitive dysfunction in patients with aluminum hydroxide-induced macrophagic myofasciitis (MMF). J Inorg Biochem (2011) 105:1457–63. 10.1016/j.jinorgbio.2011.08.006 22099155

[52] Jason A Benton C Valentine L Johnson A Torres-Harding S. The economic impact of ME/CFS: individual and societal costs. Dyn Med (2007) 7:6. 10.1186/1476-5918-7-6 18397528 PMC2324078

[53] Hickie I Davenport T Wakefield D Vollmer-Conna U Cameron B Vernon SD Post-infective and chronic fatigue syndromes precipitated by viral and non-viral pathogens: prospective cohort study. BMJ (2006) 333(7568):575. 10.1136/bmj.38933.585764.AE 16950834 PMC1569956

[54] Landay AL Jessop C Lennette ET Levy JA Chronic fatigue syndrome: clinical condition associated with immune activation. Lancet (1991) 338:707–12 10.1016/0140-6736(91)91440-6 1679864

[55] Chang CM Warren JL Engels EA. Chronic fatigue syndrome and subsequent risk of cancer among elderly US adults. Cancer (2012) 118(23):5929–36. 10.1002/cncr.27612 22648858 PMC3434293

[56] Tomljenovic L. Aluminum and Alzheimer’s disease: after a century of controversy, is there a plausible link? J Alzheimer Dis (2011) 23:567–98. 10.3233/JAD-2010-101494 21157018

[57] Rosenblum H Shoenfeld Y Amital H. The common immunogenic etiology of chronic fatigue syndrome: from infections to vaccines via adjuvants to the ASIA syndrome. Infect Dis Clin North Am (2011) 25(4):851–63. 10.1016/j.idc.2011.07.012 22054760

[58] Lee SH. Detection of human papillomavirus (HPV) L1 gene DNA possibly bound to particulate aluminum adjuvant in the HPV vaccine Gardasil. J Inorg Biochem (2012) 117:85–92. 10.1016/j.jinorgbio.2012.08.015 23078778

[59] Offit PA Jew RK. Addressing parents’ concerns: do vaccines contain harmful preservatives, adjuvants, additives, or residuals? Pediatrics (2003) 112(6):1394–7. 10.1542/peds.112.6.1394 14654615

[60] Rinaldi M Perricone R Blank M Perricone C Shoenfeld Y. Anti-*Saccharomyces cerevisiae* autoantibodies in autoimmune diseases: from bread baking to autoimmunity. Clin Rev Allergy Immunol (2013) 45(2):152–61. 10.1007/s12016-012-8344-9 23292495

[61] Lopez S Pelaez A Navarro LA Montesinos E Morales C Carda C Aluminum allergy in patients hyposensitized with aluminum-precipitated antigen extracts. Contact Dermatitis (1994) 31:37–40 10.1111/j.1600-0536.1994.tb01903.x 7924292

[62] Bergfors E Björkelund C Trollfors B. Nineteen cases of persistent pruritic nodules and contact allergy to aluminum after injection of commonly used aluminum-adsorbed vaccines. Eur J Pediatr (2005) 164(11):691–7. 10.1007/s00431-005-1704-1 16044278

[63] Falta MT Pinilla C Mack DG Tinega AN Crawford F Giulianotti M Identification of beryllium-dependent peptides recognized by CD4+ T cells in chronic beryllium disease. J Exp Med (2013) 210(7):1403–18. 10.1084/jem.20122426 23797096 PMC3698527

[64] Wen GY Wisniewski HM. Histochemical localization of aluminum in the rabbit CNS. Acta Neuropathol (1985) 68:175–84. 10.1007/BF00690191 2417440

[65] Redhead K Quinlan GJ Das RG Gutteridge JM. Aluminum-adjuvanted vaccines transiently increase aluminum levels in murine brain tissue. Pharmacol Toxicol (1992) 70:278–80. 10.1111/j.1600-0773.1992.tb00471.x 1608913

[66] Sahin G Varol I Temizer A Benli K Demirdamar R Duru S. Determination of aluminum levels in the kidney, liver, and brain of mice treated with aluminum hydroxide. Biol Trace Elem Res (1994) 41:129–35. 10.1007/BF02917223 7946900

[67] Wang XY Yao X Wan YM Wang B Xu JQ Wen YM. Responses to multiple injections with alum alone compared to injections with alum adsorbed to proteins in mice. Immunol Lett (2012) 149:88–92. 10.1016/j.imlet.2012.11.005 23183095

[68] Drevets DA Dillon MJ Schawang JS Van Rooijen N Ehrchen J Sunderkotter C The Ly-6C^high^ monocyte subpopulation transports Listeria monocytogenes into the brain during systemic infection of mice. J Immunol (2004) 172:4418–24. 10.4049/jimmunol.172.7.4418 15034057

[69] Eugenin EA Osiecki K Lopez L Goldstein H Calderon TM Berman JW. CCL2/monocyte chemoattractant protein-1 mediates enhanced transmigration of human immunodeficiency virus (HIV)-infected leukocytes across the blood-brain barrier: a potential mechanism of HIV-CNS invasion and NeuroAIDS. J Neurosci (2006) 26:1098–106. 10.1523/JNEUROSCI.3863-05.2006 16436595 PMC6674577

[70] Petrik MS Wong MC Tabata RC Garry RF Shaw CA. Aluminum adjuvant linked to Gulf war illness induces motor neuron death in mice. Neuromolecular Med (2007) 9(1):83–100. 10.1385/NMM:9:1:83 17114826

[71] Shaw CA Petrik MSJ. Aluminum hydroxide injections lead to motor deficits and motor neuron degeneration. J Inorg Biochem (2009) 103:1555–62. 10.1016/j.jinorgbio.2009.05.019 19740540 PMC2819810

[72] Shaw CA Li Y Tomljenovic L. Administration of aluminum to neonatal mice in vaccine-relevant amounts is associated with adverse long term neurological outcomes. J Inorg Biochem (2013) 128:237–44. 10.1016/j.jinorgbio.2013.07.022 23932735

[73] Tomljenovic L Shaw CA. Aluminum vaccine adjuvants: are they safe? Curr Med Chem (2011) 18:2630–7. 10.2174/092986711795933740 21568886

[74] van Rensburg SJ Potocnik FC Kiss T Hugo F van Zijl P Mansvelt E Serum concentrations of some metals and steroids in patients with chronic fatigue syndrome with reference to neurological and cognitive abnormalities. Brain Res Bull (2001) 55(2):319–25. 10.1016/S0361-9230(01)00478-6 11470334

[75] Exley C Mamutse G Korchazhkina O Pye E Strekopytov S Polwart A Elevated urinary excretion of aluminum and iron in multiple sclerosis. Mult Scler (2006) 12(5):533–40. 10.1177/1352458506071323 17086897

[76] Hernán MA Jick SS Olek MJ Jick H. Recombinant hepatitis B vaccine and the risk of multiple sclerosis: a prospective study. Neurology (2004) 63(5):838–42. 10.1212/01.WNL.0000138433.61870.82 15365133

[77] Mikaeloff Y Caridade G Suissa S Tardieu M Hepatitis B vaccine and the risk of CNS inflammatory demyelination in childhood. Neurology (2009) 72(10):873–80 10.1212/01.wnl.0000335762.42177.07 18843097

[78] Authier FJ Cherin P Creange A Bonnotte B Ferrer X Abdelmoumni A Central nervous system disease in patients with macrophagic myofasciitis. Brain (2001) 124(5):974–83. 10.1093/brain/124.5.974 11335699

[79] Shoenfeld Y Agmon-Levin N ‘ASIA’ – autoimmune/inflammatory syndrome induced by adjuvants. J Autoimmun (2011) 36(1):4–8 10.1016/j.jaut.2010.07.003 20708902

[80] Hotopf M David A Hull L Ismail K Unwin C Wessely S. Role of vaccinations as risk factors for ill health in veterans of the Gulf war: cross sectional study. BMJ (2000) 320:1363–7. 10.1136/bmj.320.7246.1363 10818024 PMC27378

[81] Cherry N Creed F Silman A Dunn G Baxter D Smedley J Health and exposures of United Kingdom Gulf war veterans. Part II: the relation of health to exposure. Occup Environ Med (2001) 58:299–306. 10.1136/oem.58.5.299 11303078 PMC1740138

[82] Theeler BJ Simper NB Ney JP. Polyglandular autoimmunity with macrophagic myofasciitis. Clin Rheumatol (2008) 27(5):667–9. 10.1007/s10067-007-0793-9 18180978

[83] Asa PB Cao Y Garry RF Antibodies to squalene in Gulf war syndrome. Exp Mol Pathol (2000) 68(1):55–64 10.1006/exmp.1999.2295 10640454

[84] Gherardi RK. Lessons from macrophagic myofasciitis: towards definition of a vaccine adjuvant-related syndrome. Rev Neurol (Paris) (2003) 159(2):162–4. 12660567

[85] Miyoshi K Miyamura T Kobayashi Y Itakura T Nishijo K Hypergammaglobulinemia by prolonged adjuvanticity in man disorders developed after augmentation mammoplasty. Jpn Med J (1964) 2122:9–14.

[86] Shoaib BO Patten BM. Human adjuvant disease: presentation as a 414 multiple sclerosis-like syndrome. South Med J (1996) 89:179–88. 10.1097/00007611-199602000-00005 8578347

[87] Luján L Pérez M Salazar E Álvarez N Gimeno M Pinczowski P Autoimmune/autoinflammatory syndrome induced by adjuvants (ASIA syndrome) in commercial sheep. Immunol Res (2013) 56(2–3):317–24. 10.1007/s12026-013-8404-0 23579772

[88] Vasey FB Zarabadi SA Seleznick M Ricca L Where there’s smoke there’s fire: the silicone breast implant controversy continues to flicker: a new disease that needs to be defined. J Rheumatol (2003) 30(10):2092–4. 14528500

[89] Guis S Pellissier JF Nicoli F Reviron D Mattei JP Gherardi RK HLA-DRB1*01 and macrophagic myofasciitis. Arthritis Rheum (2002) 46:2535–7 10.1002/art.10465 12355502

[90] Nyquist P Zhang J De Graba TJ. The -928 G/C and -362 G/C single-nucleotide polymorphisms in the promoter of MCP1: increased transcriptional activity and novel binding sites. Cerebrovasc Dis (2010) 29:242–7. 10.1159/000267849 20029197

[91] Sridhar S Botbol Y Macian F Cuervo AM. Autophagy and disease: always two sides to a problem. J Pathol (2012) 226:255–73. 10.1002/path.3025 21990109 PMC3996449

[92] Brest P Lapaquette P Souidi M Lebrigand K Cesaro A Vouret-Craviari V A synonymous variant in IRGM alters a binding site for miR-196 and causes deregulation of IRGM-dependent xenophagy in Crohn’s disease. Nat Genet (2011) 43(3):242–5. 10.1038/ng.762 21278745

[93] Stern ST Adiseshaiah PP Crist RM. Autophagy and lysosomal dysfunction as emerging mechanisms of nanomaterial toxicity. Part Fibre Toxicol (2012) 9:20. 10.1186/1743-8977-9-20 22697169 PMC3441384

[94] Zheng YT Shahnazari S Brech A Lamark T Johansen T Brumell JH. The adaptor protein p62/SQSTM1 targets invading bacteria to the autophagy pathway. J Immunol (2009) 183(9):5909–16. 10.4049/jimmunol.0900441 19812211

[95] Lee HS Daniels BH Salas E Bollen AW Debnath J Maregeta M. Clinical utility of LC3 and p62 immunohistochemistry in diagnosis of drug-induced autophagic vacuolar myopathies: a case-control study. PLoS One (2012) 7(4):e36221. 10.1371/journal.pone.0036221 22558391 PMC3338695

